# Changes of the vaginal microbiota in HPV infection and cervical intraepithelial neoplasia: a cross-sectional analysis

**DOI:** 10.1038/s41598-022-06731-5

**Published:** 2022-02-18

**Authors:** Wenyu Lin, Qiaoyu Zhang, Yaojia Chen, Binhua Dong, Huifeng Xue, Huifang Lei, Yanfang Lu, Xufang Wei, Pengming Sun

**Affiliations:** 1 Laboratory of Gynecologic Oncology, Department of Gynecology, Fujian Maternity and Child Health Hospital, Affiliated Hospital of Fujian Medical University, No. 18 Daoshan Road., Fuzhou, 350001 Fujian People’s Republic of China; 2 Fujian Key Laboratory of Women and Children’s Critical Diseases Research, Fujian Maternity and Child Health Hospital (Fujian Women and Children’s Hospital), Fuzhou, People’s Republic of China; 3 Department of Gynecology, Military Hospital of the 73rd Army Group, Xiamen, People’s Republic of China; 4 Fujian Provincial Cervical Disease Diagnosis and Treatment Health Center, Fujian Maternity and Child Health Hospital, Affiliated Hospital of Fujian Medical University, Fuzhou, Fujian People’s Republic of China; 5 Department of Clinical Laboratory, Fujian Maternity and Child Health Hospital, Affliated Hospital of Fujian Medical University, Fuzhou, Fujian People’s Republic of China; 6 Department of Gynecology, Fujian Maternity and Child Health Hospital, Affiliated Hospital of Fujian Medical University, No. 18 Daoshan Road., Fuzhou, 350001 Fujian People’s Republic of China

**Keywords:** Cancer, Microbiology, Oncology

## Abstract

This study aimed to explore the changes of the vaginal microbiota and enzymes in the women with high-risk human papillomavirus (HR-HPV) infection and cervical lesions. A total of 448 participants were carried out HPV genotyping, cytology tests, and microecology tests, and 28 participants were treated as sub-samples, in which vaginal samples were characterized by sequencing the bacterial 16S V4 ribosomal RNA (rRNA) gene region. The study found the prevalence of HR-HPV was higher in patients with BV (*P* = 0.036). The HR-HPV infection rate was 72.73% in *G. vaginalis* women, which was significantly higher than that of women with *lactobacillus* as the dominant microbiota (44.72%) (*P* = 0.04). The positive rate of sialidase (SNA) was higher in women with HR-HPV infection (P = 0.004) and women diagnosed with cervical intraepithelial neoplasia (CIN) (*P* = 0.041). In HPV (+) women, the α-diversity was significantly higher than that in HPV (−) women. The 16S rRNA gene-based amplicon sequencing results showed that *Lactobacillus* was the dominant bacteria in the normal vaginal microbiota. However, the proportion of *Gardnerella* and *Prevotella* were markedly increased in HPV (+) patients. *Gardnerella* and *Prevotella* are the most high-risk combination for the development of HPV (+) women. The SNA secreted by *Gardnerella* and *Prevotella* may play a significant role in HPV infection progress to cervical lesions.

## Introduction

Human papillomavirus (HPV) infections gave rise to over 600,000 cases of cancer in a year^1^. Most women will have been infected with HPV by intercourse during their lives, most HPV infections fade away by themselves in a few months, a few HPV infections persist and cause lesions^2^. Although most instantaneous HPV infections are cleared by the immune system, persistent infections can cause viral gene integration into the host genome and lead to HPV-related cancer^3^. Previous studies have found that local microbiota, epithelial surface integrity, immune regulation were synergistic factors in the progression of HPV to cancer. Nevertheless, little is known about the functional composition of the local microbiota and how it varies by cervicovaginal syndromes, infections, and diseases^4^.

The vaginal microenvironment can be categorized into five kinds of community state types (CSTs), *Lactobacillus* spp. was the dominant microbiota in CST I, II, III, and V. And the CSTs have different *Lactobacillus* species types, such as *Lactobacillus-crispatus, Lactobacillus-iners, Lactobacillus-jensenii, Lactobacillus-gasseri,* and so on^5^. Notably, The features of CST IV are higher vaginal pH (> 4.5), lack of *Lactobacillus*, and abundance of *Gardnerella*. These features are also features of bacterial vaginosis (BV)^6^. BV is a kind of mixed infection characterized by the reduction of *Lactobacillus* and the multiplication of pathogen, mainly *Gardnerella vaginalis* (*G. vaginalis*), accompanied by increased vaginal pH. It has been reported that BV infection in the Chinese population ranges from 10.5 to 51.6%^7^. Previous studies indicate that there is a close correlation between BV infection and HR-HPV persistence. A study by Gillet^8^ showed that patients with BV are more prone to HR-HPV infections, while Guo^9^ further found that a BV infection prolongs the duration and the regression time of HR-HPV infections.

As the main pathogen of BV, the detection rate of *Gardnerella* was significantly increased in HR-HPV-positive women^10^. In a 2-year longitudinal prospective study, the presence of specific anaerobic groups, including *Gardnerella*, was associated with the persistence and slow degradation of CIN2^11^. Meanwhile, different dominant bacterial communities produce different metabolomes. *Gardnerella* secretes SNA while elevated SNA concentration was associated with increased risk for cervical lesion^12^. The vaginal metabolome of HPV (−) women differed from HPV (+) women in terms of several metabolites, including biogenic amines, glutathione, and lipid-related metabolites^13^. *L. crispatus* producing hydrogen peroxide (H_2_O_2_) show the strongest associations with vaginal health and are depleted in dysbiosis^14^. With the development of high-throughput sequencing, there is still a lack of systematic and comprehensive studies to investigate the types and enzymes of vaginal microbiota and their relationship with HPV infection and cervical lesions.

To solve the above problems, this study started from clinical data, with the help of high throughput sequencing, aimed to explore the changes of the vaginal microbiota and enzymes in the HR-HPV infection progress to cervical lesions and provide ideas for further exploration of the interaction mechanism between vaginal microbiota and HPV infection.

## Results

### Socio-demographic characteristics of participants

Characteristics of 448 participants were analyzed. Participants with normal cervical pathology or negative cytology and HPV (−) were defined as the normal group. Our study indicated that there were no significant differences in terms of age, nationality, marital status, and reproductive history among the groups (P > 0.05; Table 1).

**Table 1:** Socio-demographic characteristics among participants.

variables	Normal group (n = 379) n (%)	CIN1+ (n = 51) n (%)	P value
**Age (years)**			
(Mean, SD)	36.6 ± 9.3	39.5 ± 11.1	0.115^†^
**Nation**			
Han nationality	369 (97.36)	48 (94.12)	0.191^§^
Other nationality	10 (2.64)	3 (5.89)	
**Marital status**			
Married	241 (63.59)	27 (52.94)	0.141
Unmarried	138 (36.41)	24 (47.06)	
**Reproductive history**			
Yes	227 (59.89)	26 (50.98)	0.225
No	152 (40.11)	25 (49.02)	
**Postoperative**			
Yes	126 (33.25)	51 (100.00)	
No	253 (66.75)	0 (0.00)	

^†^Is a non-parametric test.

^§^Is Fisher’s exact probability method, the rest are all using chi-square test; the lesions group includes patients with CIN I, CIN II, CIN III and cervical cancer. The normal group is the patients with normal cervical pathology or negative TCT and HPV results.

### Distribution of HPV genotypes in patients with different cervical intraepithelial neoplasias

The HR-HPV infection rates in women with NILM and CIN were 22.17% (88/397) and 72.55% (37/51), and the difference was statistically significant (P < 0.001). Among all participants, the most susceptible HPV types were HPV52 (6.7%), HPV58 (4.7%), and HPV16 (4.2%). Among the women diagnosed with NILM, the most susceptible HPV types were HPV52 (5.5%), HPV58 (2.9%), and HPV51 (2.9%). Among women diagnosed with CIN I, the most susceptible HPV genotypes were HPV52 (27.3%), HPV16 (18.2%), and HPV18 (18.2%). The most susceptible HPV types were HPV58 (20.7%), HPV16 (17.2%), and HPV18 (10.3%) in patients diagnosed with CIN II and above. The detailed results are shown in Table 2.

**Table 2:** Distribution characteristics of HPV.

HPV type	NILM N = 379 (%)	CIN I N = 22 (%)	≥ CIN II N = 29 (%)	Total N = 430 (%)
**High risk**				
HPV16	9 (2.4)	4 (18.2)	5 (17.2)	18 (4.2)
HPV18	10 (2.6)	4 (18.2)	3 (10.3)	17 (4.0)
HPV31	4 (1.1)	0 (0.0)	0 (0.0)	4 (0.9)
HPV33	3 (0.8)	0 (0.0)	2 (6.9)	5 (1.2)
HPV35	4 (1.1)	0 (0.0)	1 (3.4)	5 (1.2)
HPV39	4 (1.1)	2 (9.1)	0 (0.0)	6 (1.4)
HPV45	1 (0.3)	1 (4.5)	1 (3.4)	3 (0.7)
HPV51	11 (2.9)	2 (9.1)	2 (6.9)	15 (3.5)
HPV52	21 (5.5)	6 (27.3)	2 (6.9)	29 (6.7)
HPV53	9 (2.4)	1 (4.5)	2 (6.9)	12 (2.8)
HPV56	6 (1.6)	2 (9.1)	1 (3.4)	9 (2.1)
HPV58	11 (2.9)	3 (13.6)	6 (20.7)	20 (4.7)
HPV59	5 (1.3)	2 (9.1)	0 (0.0)	7 (1.6)
HPV66	2 (0.5)	1 (4.5)	1 (3.4)	4 (0.9)
HPV68	2 (0.5)	0 (0.0)	2 (6.9)	4 (0.9)
HPV73	4 (1.1)	0 (0.0)	0 (0.0)	4 (0.9)
HPV82	0 (0.0)	0 (0.0)	2 (6.9)	2 (0.5)
**Low risk**				
HPV6	4 (1.1)	1 (4.5)	0 (0.0)	5 (1.2)
HPV11	0 (0.0)	1 (4.5)	0 (0.0)	1 (0.2)
HPV42	9 (2.4)	1 (4.5)	1 (3.4)	11 (2.6)
HPV43	2 (0.5)	1 (4.5)	0 (0.0)	3 (0.7)
HPV81	10 (2.6)	2 (9.1)	2 (6.9)	14 (3.3)

### The relationship between vaginal microenvironment and HPV infection, cervical lesions

In this study, the infection rate of BV was the highest, accounting for 41.96% (188/448), followed by vulvovaginal candidiasis (VVC), aerobic vaginitis (AV), cytolytic vaginosis (CV), and trichomonal vaginitis (TV), accounting for 12.05% (54/448), 5.13% (23/448) and 1.78% (8/448) and 0.89% (4/448). BV and HR-HPV infections were associated with each other. The difference was statistically significant (P = 0.036). The HR-HPV infection rate was 72.73% in *G. vaginalis* women, which was significantly higher than that of women with *lactobacillus* as the dominant microbiota (44.72%) (*P* = 0.04). However, no obvious correlation with HR-HPV infection was found between other dominant microbiota. We also found that the positive rate of SNA was higher in HR-HPV infection women (P = 0.004). The results are shown in Table 3.

**Table 3:** The changes of micro-environment factors between different HR-HPV infection women.

Variables	HR-HPV		χ^2^	P
	Positive (n = 138)	Negative (n = 310)		
**pH**				
Normal (n = 197)	64 (32.49)	133 (67.51)	0.468	0.494
Abnormal (n = 251)	74 (29.48)	177 (70.52)		
**Microbiome density**				
Normal (n = 332)	91 (27.41)	241 (72.59)	0.420	0.517
Abnormal (n = 143)	47 (32.87)	96 (67.13)		
**Microbiome diversity**				
Normal (n = 405)	128 (31.60)	277 (68.40)	1.271	0.260
Abnormal (n = 43)	10 (23.26)	33 (76.74)		
**Dominant bacteria**				
G + (n = 233)	72 (30.90)	161 (69.09)	–	–
G − (n = 183)	59 (32.24)	124 (67.76)	0.085	0.770
GV (n = 11)	8 (72.73)	3 (27.27)	8.340	0.040
Other G − (n = 172)	51 (29.65)	121 (70.35)	0.070	0.790
Other (n = 32)	7 (21.88)	25 (78.13)	1.090	0.290
**BV**				
Positive (n = 188)	68 (36.17)	120 (63.83)	4.377	0.036
Negative (n = 260)	70 (26.92)	190 (73.08)		
**AV**				
Positive (n = 23)	6 (26.09)	17 (73.91)	0.253	0.615
Negative (n = 425)	132 (31.06)	293 (68.94)		
**VVC**				
Positive (n = 54)	18 (33.33)	36 (66.67)	0.184	0.668
Negative (n = 394)	120 (30.46)	274 (69.54)		
**TV**				
Positive (n = 4)	0 (0.00)	4 (100.00)	–	0.317§
Negative (n = 444)	138 (30.80)	306 (69.20)		
**CV**				
Positive (n = 8)	2 (25.00)	6 (75.00)	–	> 0.999^§^
Negative (n = 440)	136 (30.91)	304 (69.09)		
**Leukocyte esterase**				
Positive (n = 348)	107 (30.75)	241 (69.25)	0.002	0.960
Negative (n = 100)	31 (31.00)	69 (69.00)		
**Sialidase**				
Positive (n = 84)	37 (44.05)	47 (55.95)	8.508	0.004
Negative (n = 364)	101 (27.75)	263 (72.25)		
**Catalase**				
Positive (n = 370)	109 (29.46)	261 (70.54)	1.800	0.180
Negative (n = 78)	29 (37.18)	49 (62.82)		

Normal pH values between 3.8–4.5, and the rest are regarded as abnormal pH values. The normal flora diversity is the flora diversity ++  to  +++, the normal flora density is the flora density ++  to  +++, the rest is regarded as the flora diversity and the flora density abnormality.

*G*+ Gram-positive bacteria, *G− *Gram-negative bacteria, *GV* Gardnerella vaginalis, *AV* aerobic vaginitis, *BV* bacterial vaginosis, *CV* cytolytic vaginosis, *TV* trichomonas vaginitis, *VVC* vulvovaginal candidiasis.

^§^Is Fisher’s exact test method.

Among the 448 participants in this study, 18 were excluded because they did not undergo cytological or pathological examination. The remaining participants were in a ratio of approximately 1:2 according to HR-HPV infection or not. The pathological results of NILM and ≥ CIN I patients were matched, and they were divided into a case group (≥ CIN I) and a control group. Ultimately, a total of 177 patients were included in the second part of this study, including 51 cases and 126 controls. In women diagnosed with CIN, the positive rate of SNA increased (31.37% vs 17.46%, P = 0.041). However, catalase or leukocyte esterase (LE) were not significantly associated with cervical lesions (Table 4).

**Table 4:** The changes of micro-environment factors between different cervical lesions.

Variables	Cervical cytology				P	Histology		P
	NILM (n = 126)	ASCUS (n = 25)	LSIL (n = 18)	HSIL (n = 8)		Normal (n = 126)	CIN1 + (n = 51)	
**pH**								
Normal (n = 80)	56 (70.00)	13 (16.25)	9 (11.25)	2 (2.50)	0.586	55 (68.75)	25 (31.25)	0.516
Abnormal (n = 97)	70 (72.16)	12 (12.37)	9 (9.28)	6 (6.19)		71 (73.20)	26 (26.80)	
**Microbiome density**								
Normal (n = 120)	84 (70.00)	19 (15.83)	13 (10.83)	4 (3.33)	0.549	83 (69.17)	37 (30.83)	0.389
Abnormal (n = 57)	42 (73.68)	6 (10.53)	5 (8.77)	4 (7.02)		43 (75.44)	14 (24.56)	
**Microbiome diversity**								
Normal (n = 161)	114 (70.81)	23 (14.29)	17 (10.56)	7 (4.35)	0.918	115 (71.43)	46 (28.57)	0.779
Abnormal (n = 16)	12 (75.00)	2 (12.50)	1 (6.25)	1 (6.25)		11 (68.75)	5 (31.25)	
**Dominant bacteria**								
G+ (n = 93)	64 (68.82)	15 (16.13)	11 (11.83)	3 (3.23)	–	65 (69.89)	28 (30.11)	–
G− (n = 73)	54 (73.97)	9 (12.33)	7 (9.59)	3 (4.11)	0.830	54 (73.97)	19 (26.03)	0.562
GV (n = 8)	5 (62.50)	0 (0.00)	0 (0.00)	3 (37.50)	0.015	4 (50.00)	4 (50.00)	0.259
Other G − (n = 65)	49 (75.38)	9 (13.85)	7 (10.77)	0 (0.00)	0.578	50 (76.92)	16 (23.08)	0.415
Other (n = 11)	8 (72.73)	1 (9.09)	0 (0.00)	2 (18.18)	0.134	7 (63.64)	4 (36.36)	0.734
**BV**								
Positive (n = 81)	58 (71.60)	10 (12.35)	8 (9.88)	5 (6.17)	0.765	58 (71.60)	23 (28.4)	0.910
Negative (n = 96)	68 (70.83)	15 (15.63)	10 (10.42)	3 (3.13)		68 (70.83)	28 (29.17)	
**AV**								
Positive (n = 7)	6 (85.71)	0 (0.00)	1 (14.29)	0 (0.00)	0.757	4 (57.14)	3 (42.86)	0.413
Negative (n = 170)	120 (70.59)	25 (14.71)	17 (10.00)	8 (4.71)		122 (71.76)	48 (28.24)	
**VVC**								
Positive (n = 22)	16 (72.73)	3 (13.64)	2 (9.09)	1 (4.55)	> 0.999	17 (77.27)	5 (22.73)	0.501
Negative (n = 155)	110 (70.97)	22 (14.19)	16 (10.32)	7 (4.52)		109 (70.32)	46 (29.68)	
**TV**								
Positive (n = 2)	2 (100.00)	0 (0.00)	0 (0.00)	0 (0.00)	> 0.999	1 (50.00)	1 (50.00)	0.494
Negative (n = 175)	124 (70.86)	25 (33.33)	18 (10.29)	8 (4.57)		125 (71.43)	50 (28.57)	
**CV**								
Positive (n = 3)	3 (100.00)	0 (0.00)	0 (0.00)	0 (0.00)	> 0.999	2 (66.67)	1 (33.33)	> 0.999
Negative (n = 174)	123 (70.69)	25 (14.37)	18 (10.34)	8 (4.60)		123 (70.69)	50 (28.74)	
**Leukocyte esterase**								
Positive (n = 134)	98 (73.13)	17 (12.69)	12 (8.96)	7 (5.22)	0.508	96 (71.64)	38 (28.36)	0.813
Negative (n = 43)	28 (65.12)	8 (18.60)	6 (13.95)	1 (2.33)		30 (69.77)	13 (30.23)	
**Sialidase**								
Positive (n = 38)	25 (65.79)	4 (10.53)	6 (15.79)	3 (7.89)	0.312	22 (57.89)	16 (42.11)	0.041
Negative (n = 139)	101 (72.66)	21 (15.11)	12 (8.63)	5 (3.60)		104 (74.82)	35 (25.18)	
**Catalase**								
Positive (n = 140)	101 (72.14)	20 (14.29)	16 (11.43)	3 (2.14)	0.042	103 (73.57)	37 (26.43)	0.220
Negative (n = 37)	25 (67.57)	5 (13.51)	2 (5.41)	5 (13.51)		23 (62.16)	14 (37.84)	

HSIL includes HSIL, AGC, ASC-H. The above results all use Fisher’s exact test method.

*G*+ Gram-positive bacteria, *G− *Gram-negative bacteria, *GV* Gardnerella vaginalis, *AV* aerobic vaginitis, *BV* bacterial vaginosis, *CV* cytolytic vaginosis, *TV* trichomonas vaginitis, *VVC* vulvovaginal candidiasis.

### Changes of vaginal microbial diversity and microbiota in HPV infection women

**Figure 1:**
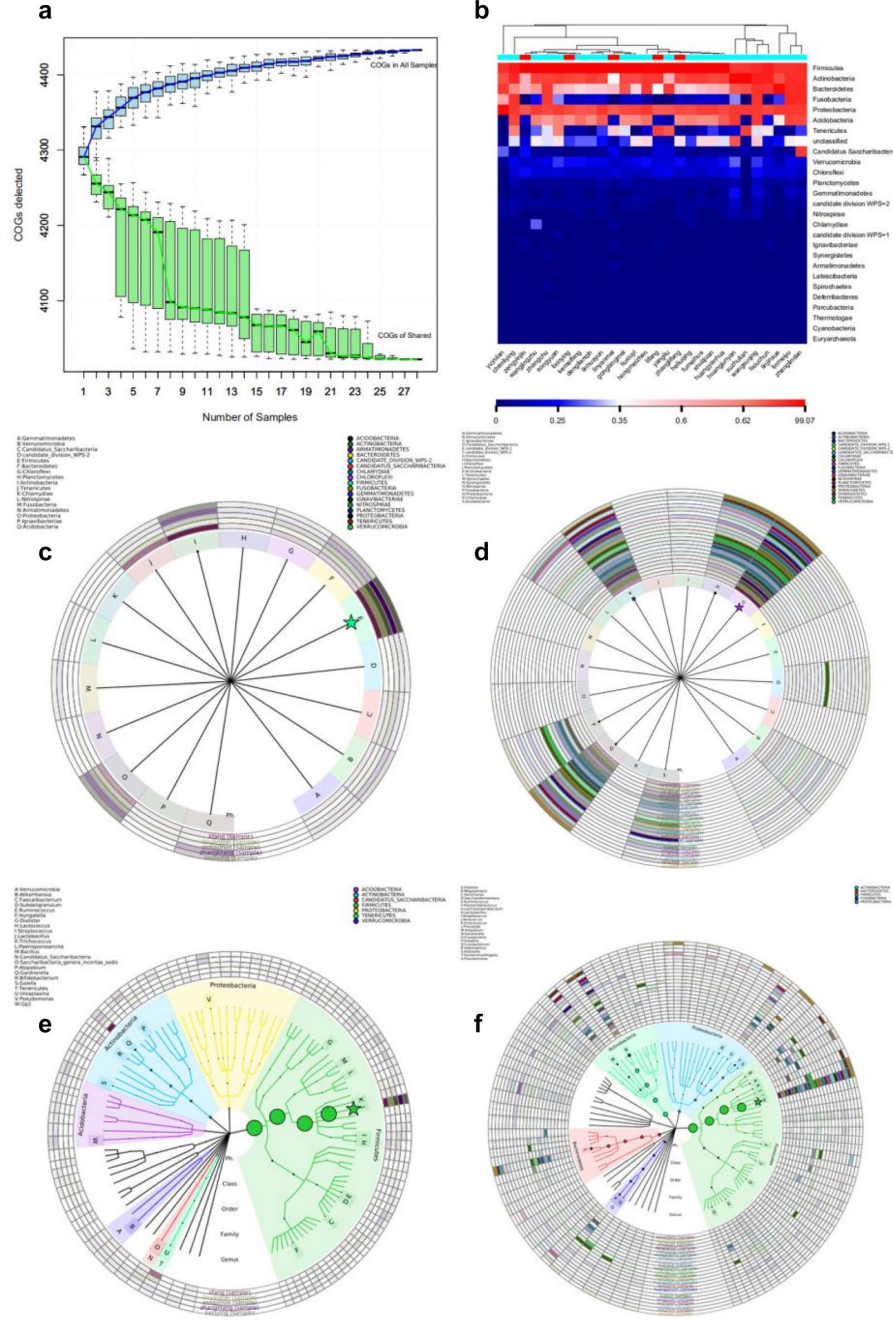
(**a**) COG function cumulative curve: The species accumulation curve can be used to judge whether the sample quantity is sufficient. The sharp rise of the curve indicates that the sample quantity is insufficient and the sampling quantity should be increased. When the curve flattens out, it indicates that the sampling is sufficient for data analysis. (**b**) Species abundance heat map in the phylum horizontal: species abundance heat map, drawn with a species abundance matrix, each column in the figure represents a sample, the row represents the community structure, the color block represents the relative species abundance value, the redder the color, the higher the relative abundance. (**c–f**) Visualization of classification and phylogeny information drawn by GraPhlAn and iTOL, According to the taxonomic comparison results of each sample, the dominant species were selected, and the species abundance information was combined to display in a ring-shaped tree diagram. (**c**) The visualization of the phylum level in HPV-negative group. (**d**) The visualization of the phylum level in HPV-positive group. (**e**) The visualization of the genus level in HPV-negative group. (**f**) The visualization of the genus level in HPV-positive group.

In our study, 28 samples were carried out high-throughput sequencing. Twenty-three HPV (+) and five HPV (−) samples were collected for the study and the control groups, respectively. There was no significant difference in age between HPV (+) and HPV (−) groups (P = 0.666). The curve plateaued (Fig. 1a,b) when the sample size was approximately 20, indicating that although 28 cases seem small, it was enough for data analysis. Therefore, the sample size was ample in this study. The evolutionary classification tree of the top 100 species with their abundance, and the corresponding phylum or genus of the top 20 species with their abundance (marked with asterisks) are marked in different colors (Fig. 1c–f).

**Figure 2:**
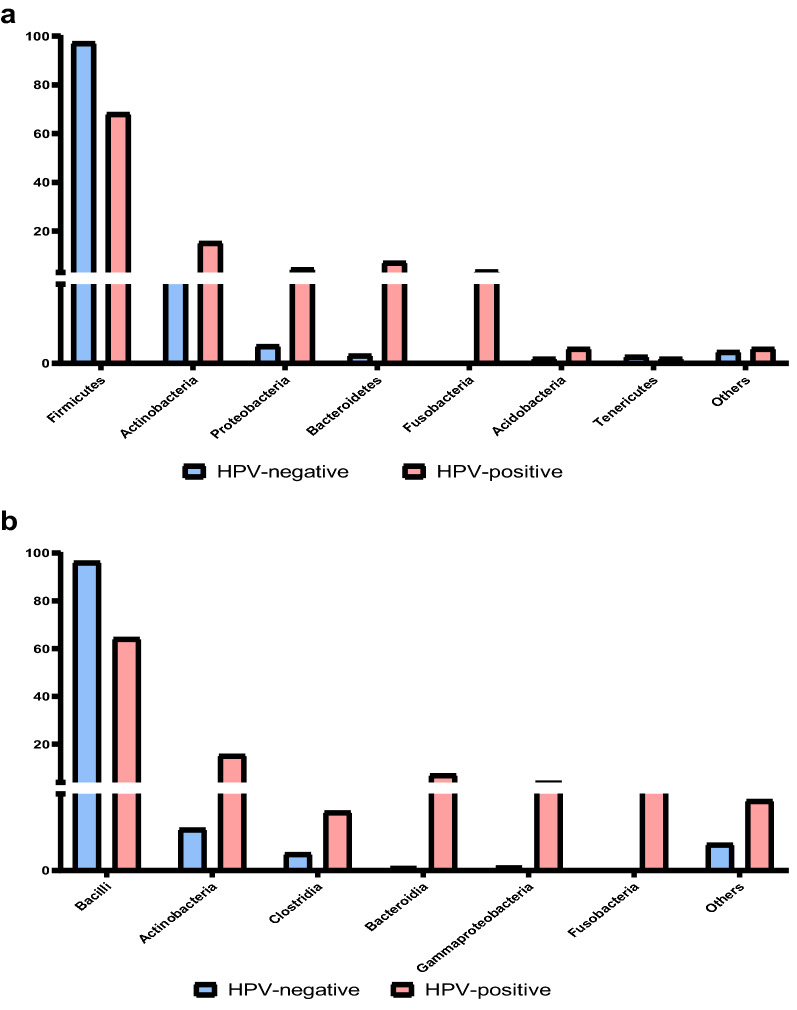

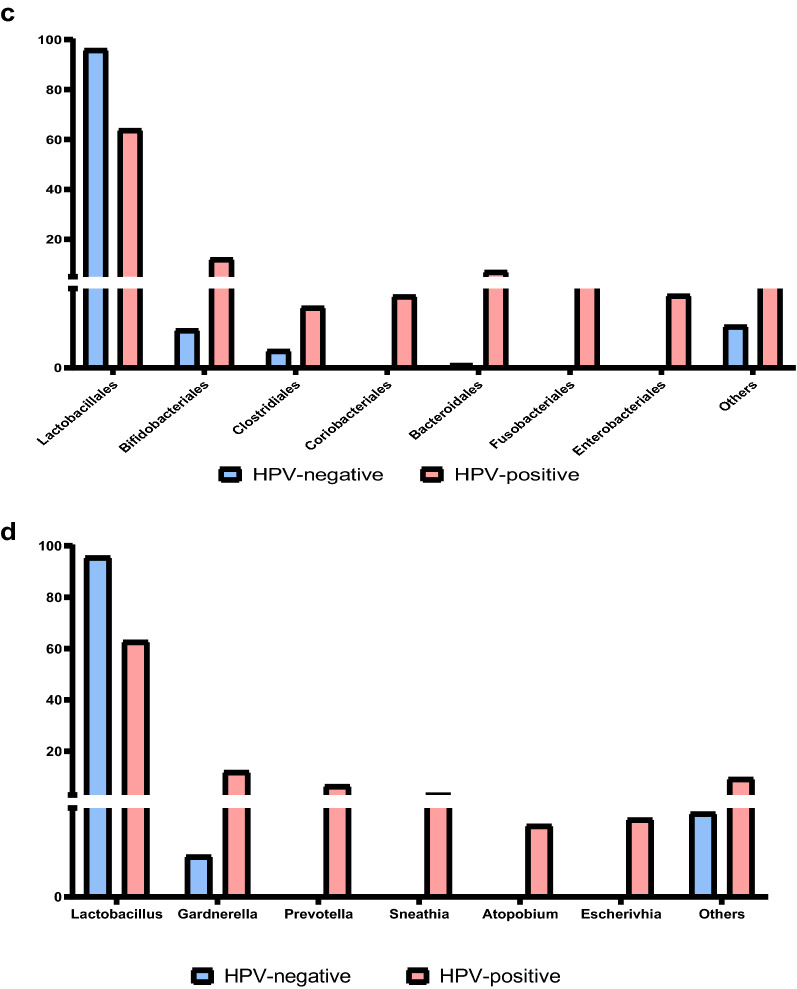
Vaginal microbiota distribution in HPV infectious women. (**a**) Vaginal microbiota in the phylum level of the HPV-negative group and the HPV-positive group. (**b**) Vaginal microbiota in the class level of the HPV-negative group and the HPV-positive group. (**c**) Vaginal microbiota in the order level of the HPV-negative group and the HPV-positive group. (**d**) Vaginal microbiota in the genus level of the HPV-negative group and the HPV-positive group.

The distribution of phylum, class, order and genus of vaginal microbiota in HPV infected women were shown in Fig. 2. Most of the vaginal microbial communities in the overall samples belong to the following phylum: *Firmicutes, Actinobacteria, Bacteroidetes, Fusobacteria, Proteobacteria* (Fig. 2a). *Firmicutes* accounted for 97.38% among total microbiota, which was the main vaginal microbiota in the HPV (−) women. However, the proportion of *Firmicutes* decreased (68.26%), and the proportions of *Actinobacteria* and *Bacteroides* increased (respectively 15.45%, 4.58%) in the HPV (+) women. The composition and differences of vaginal microbiota between the two groups were further analyzed at the genus level (Fig. 2d). In the HPV (−) women, *Lactobacillus* was the dominant bacteria (95.73%), with a small amount of *Gardnerella* (1.39%), *Atopobium* (0.03%) and other genera. In the HPV (+) women, the composition of the vaginal bacterial community structure had significant changes, mainly reflected in the decrease of *Lactobacillus* (62.91%), and the increase of *Gardnerella* (12.22%) and *Prevotella* (6.78%).

**Figure 3:**
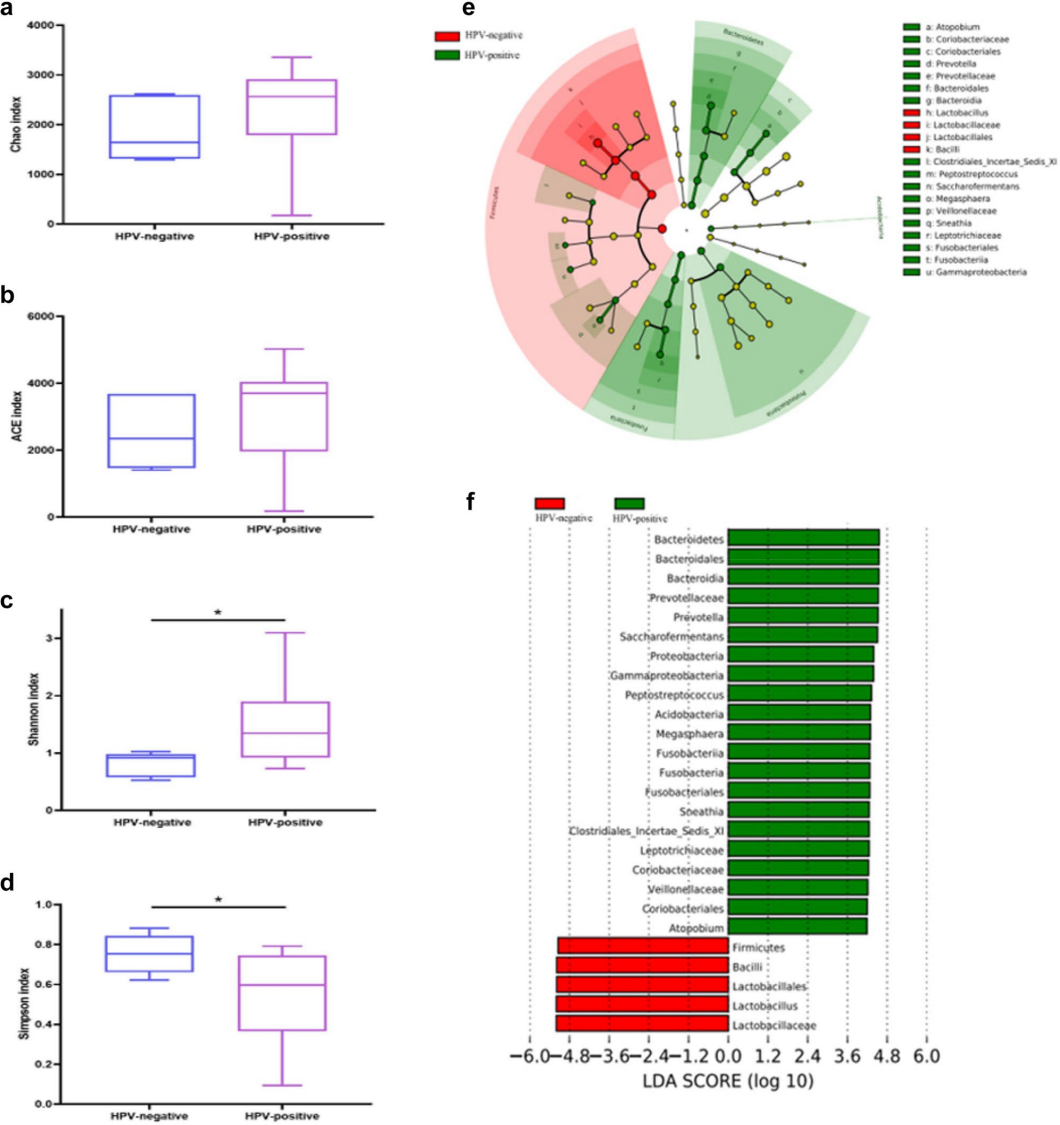
(**a–d**) Microbial α diversity between HPV-negative group and HPV-positive group. (**e**) LEfSE analysis ring tree diagram shows the two groups’ microbial distribution: nodes of different colors in the branches indicate the groups of microorganisms that play an important role in the corresponding group of the color. Yellow nodes indicate groups of microorganisms that have not played an important role. The species names represented by the English letters in the picture are shown in the legend on the right. (**f**) LDA scores distribution histogram shows the two groups’ dominant microbial distribution: the X-axis is the LDA score obtained after LDA analysis, and the Y-axis is the group of microorganisms with significant effects.

The diversity indicators of the two group samples were shown in Fig. 3a–d. In HPV (+) women, the Shannon index was higher than the HPV (−) women (F = 6.14, P = 0.023), indicating that the number of microbiota in HPV (+) women was more. In HPV (+) women, the Simpson index was lower (F = 9.494, P = 0.006), suggesting that the complexity of the vaginal microbiota was increased in patients with HPV infection and decreased in cases of vaginal health. However, the Chao index or ACE index were not related to HPV infection (P > 0.5).

To discover the changes of vaginal microbiota in HPV infection women, LEfSe analysis was performed on the two groups (Fig. 3e,f). The results showed that *Lactobacillus* was the main dominant bacteria in the HPV (−) women, and *Prevotella* was a distinct microbiota that plays a significant role in the HPV (+) women according to the Linear discriminant analysis (LDA) scores.

## Discussion

The emerging studies suggest that vaginal microenvironment plays an essential role in women’s health, specifically in sexually transmitted diseases. This is a cross-sectional study to explore the changes of the vaginal microbiota and enzymes in the HPV infection and cervical lesions. Overall, a significantly higher microbiota diversity was observed in HPV (+) women than that in HPV (−) women. The increase of *Gardnerella* and *Prevotella* and the decrease of *Lactobacillus* are closely associated with HPV infection.

Previous studies indicated *Lactobacillus* is the dominant bacteria, which play important role in protecting the health of women’s lower reproductive tract^15^. The vaginal microbiota is primarily dominated by one of the four most common *Lactobacillus* species: *Lactobacillus crispatus, Lactobacillus iners, Lactobacillus gasseri,* and *Lactobacillus jensenii*^5^*.* Lee et al.^16^ found the proportion of *Lactobacillus* was lower in HPV (+) patients. On the one hand, *Lactobacillus* maintains the weak acid environment of the vagina through its own lactic acid. On the other hand, a large quantity of *Lactobacillus* can reduce and inhibit the planting and growth of some opportunistic pathogenic bacteria to protect the lower reproductive tract from infection^17,18^. The presence of the signature “abnormal vaginal microbiota” in CIN was found by a laboratory culture in 1992 and confirmed in subsequent studies^19^. When the vagina’s protective microbiota was destroyed, the defense against pathogen infection was weakened.

In this study, We have identified vaginal microenvironment disorder was bound up with HPV infection. Compared with BV (−) women, the HR-HPV infection rate in BV (+) patients increased. BV is associated with an increased risk of detection of HPV, and HPV infections are associated with an increased risk of BV^20^. Vaginal microenvironment disturbance was associated with increased inflammatory cytokines, mucosal injury and chronic inflammation. In order to investigate the mechanism of BV and HPV infection, Rodriguez-Cerdeira et al. suggested that *G. vaginalis* is one of the common microbiota in HPV (+) women^21^, and this has been reported in other researches that using next-generation sequencing^22,23^. Women with BV had higher levels of the cytokine interleukin (IL)-1β and lower levels of IL-17^24,25^.

As an important component of vaginal microenvironment, metabolomes play an important role in the pathogenicity of microbiota. The H_2_O_2_ produced by *Lactobacillus* can catalyze peroxidase and further produce hypochlorite. It is a process that can prevent HPV from invading into cervical epithelial cells and prevent cervical lesions^26^. LE is an intracellular enzyme. When vaginal inflammation occurs, a large number of white blood cells can gather to engulf pathogens, resulting in the destruction of white cell membrane, and thus LE can be detected. However, no studies have linked LE to any type of vaginal inflammation. Similarly, this study also showed that there was no statistical significance between LE and HPV infection or cervical intraepithelial neoplasia. *Gardnerella* adheres tightly to the surface of vaginal epithelial cells, forms a dense biofilm, and can release vaginal cytolysin, which may inhibit the effect of vaginal mucosal barrier immunoglobulin A. *Gardnerella* can produce SNA, which can degrade mucosal protective factors (such as mucin) and causes vaginal epithelial cells to dissolve and expel^27^. SNA is an enzyme that cleaves terminal sialic acid residues and is associated with tissue destruction, immune response evasion, bacterial invasion, and access to bacterial-associated nutrients^28^. In addition to *Gardnerella* bacteria, such as *Prevotella* bacteria*, Bacteroides* bacteria and *Mobiluncus* bacteria also produce SNA^29^. SNA usually occupies terminal positions attached to mucosal defense factors, such as secretory IgA, secretory components, lactoferrin, and secretory leukocyte protease inhibitors^30^.

In order to investigate the mechanism of cervical lesions caused by vaginal microbiota and metabolomes, Zariffard et al. found that^31^ the expression level of Toll-like receptor—(TLR) 4 mRNA in vaginal and cervical epithelial cells was significantly increased in patients with *G. vaginalis*-infected BV. Previous studies showed that TLR9 recognizes HPV infection and initiates immune response. Experiments at the transcriptional level confirmed that E6 and E7 oncoproteins directly downregulate TLR9^32^. However, whether *Gardnerella* activates the TLRs-related pathways through SNA to cause HPV infection is still unknown and needs to be assessed. All of these mucosal, bacterial, and immune activations associated with BV and *G. vaginalis* may lead to progression of HPV infection to cervical cancer.

This study also has some limitations. First, this is a cross-sectional study to explore the changes of the vaginal microbiota and enzymes in women with HPV infection and cervical lesions, further experiments are needed to confirm that SNA secreted by *Gardnerella* and *Prevotella* contributes to HPV causing cervical lesions. Secondly, our study only focused on the distribution of different microbiota, ignoring the identification of *Lactobacillus* species. Recent researches suggest that *Lactobacillus iners* is a transitional species that colonize after the vaginal environment is disturbed and leads to BV, sexually transmitted infections, and adverse pregnancy outcomes^33^. Further studies are necessary to identify the exact role of different *Lactobacillus* species in larger samples.

Our research demonstrates vaginal microenvironment, especially BV was closely related to HPV infection. So more attention should be paid to the prevention, discovery and proper management of BV in HPV infection women. Particularly, compared to HPV (−) women, *Gardnerella* and *Prevodella* are the most high-risk combination for the development of HPV (+) women. The SNA secreted by *Gardnerella* and *Prevodella* may play a significant role in HPV infection progress to cervical lesions. This finding provides ideas for further exploration of the interaction mechanism between vaginal microbiota and HPV infection.

## Materials and methods

### Study population

**Figure 4:**
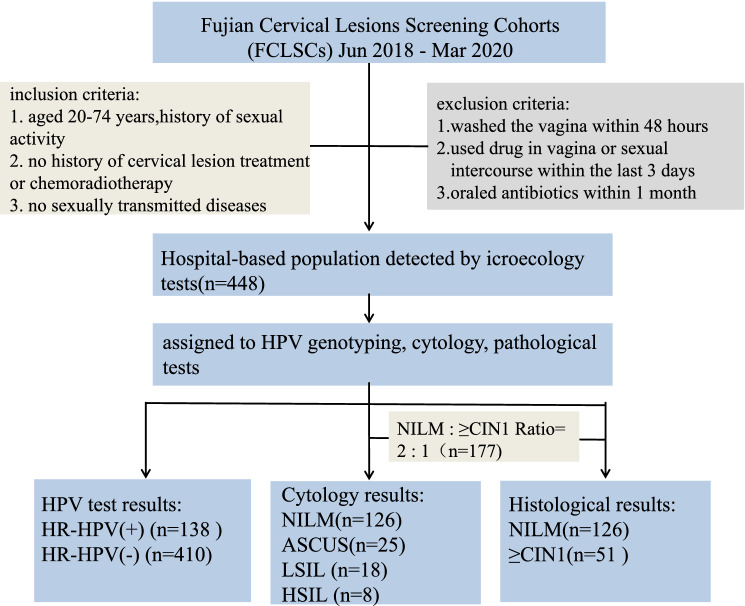
Flowchart of the study protocol.

The study participants were selected from the Fujian Cervical Lesions Screening Cohorts (FCLSCs), China. A total of 448 participants have carried out microecology tests, HPV genotyping and cytology tests, and 28 participants were treated as sub-samples, in which vaginal microecological samples were characterized by sequencing the region of bacterial 16S V4 ribosomal RNA (rRNA) gene (Fig. 4). All participants came from Fujian Maternity and Child Health Hospital, affiliated hospital of Fujian Medical University cohort which was established from June 2018 to March 2020. The participants were eligible when the following inclusion criteria were satisfied: history of sexual activity, aged 20–74 years, no history of cervical lesion treatment or chemoradiotherapy, no severe immune system diseases, no sexually transmitted diseases. The exclusion criteria are shown below: washed the vagina within 48 h, used drug in vagina or had sexual intercourse within the last 3 days, oraled antibiotics within 1 month. The Ethics Committees of the Fujian Maternity and Child Health Hospital approved this study (2020KY015), and all individuals in this study signed informed consent. All experiments in the text were carried out in compliance with the relevant rules and regulations and under the supervision and guidance of the Ethics Committee.

### Sample collection

The vaginal samples were collected from the upper third of vaginal walls by rotating for 10–15 s by study participants using cotton swabs (Santai, Jiangsu, China). The cotton swabs were inserted into a tube (Vaginal micro-microbiota diagnostic unit-700, Shtars, China) carefully avoiding skin contamination. The samples were stored at − 20 °C as soon as possible after collection for the vaginal microbiota analysis.

The researchers used plastic brushes to collect cervical cells from all participants’ cervix, elutioned in ThinPrep PreservCyt Solution (Hologic Inc., Madison, WI, USA), and stored specimens at 4 °C in laboratory immediately.

### Vaginal microbiological metabolites detection

The Vaginal secretions were obtained on 1/3 of the vaginal sidewall. Check whether there is trichomonad, mycelium, clue cells under the microscope after daubing on clean slide. H_2_O_2_, LE and SNA in secretions were detected by bPR-2014A vaginitis automatic detector and supporting detection kit (Master Biotechnology Co., Ltd, Jiangsu, China). Vaginal PH value was determined by color strips. If the PH value was no more than 4.5 (pH ≤ 4.5), the result was defined as normal. On the contrary, pH > 4.5 was defined as abnormal. Vagina cleanness was diagnosed in accordance with the standard of National Clinical Laboratory Practice Guideline 18: I–II were defined as normal vagina cleanness, and vagina cleanness III–IV defined as abnormal. AV, BV, CV, TV and VVC were all negative or positive. The Nugent scoring method was used to diagnose BV. The Nugent score was calculated by assessing the numbers of *Lactobacillus* morphotypes (scored as 0–4), *G. vaginalis* morphotypes (scored as 0–4), and *Mobiluncus* morphotypes (scored as 0–2). A Nugent score of 7–10 was interpreted as consistent with BV and a score of 4–6 as intermediate, while a score of 0–3 was interpreted as negative for BV. SNA colorless is normal (−), red or purple is positive (+). LE colorless is normal (−), and green or blue is positive (+). H_2_O_2_ > 2 mmol/L is red or purple, negative (−), H_2_O_2_ < 2 mmol/L is positive (+), blue. All laboratory procedures were conducted according to the manufacturer’s instructions.

### HPV genotyping

The HPV genotypes (16, 18, 31, 33, 35, 39, 45, 51, 52, 53, 56, 58, 59, 66, 68, 73, 82, 83, 6, 11, 42, 43, 81) were detected by Polymerase chain reaction-reverse dot blot (PCR-RDB) HPV genotyping kit (YaNeng Biosciences, Shenzhen, China)^34^. This method and kit have been approved by China Food and Drug Administration (Approval number 20020515). The procedures were conducted according to the manufacturer’s instructions.

### Liquid-based cytology

The cytological samples were blinded and independently evaluated by two experienced cytopathologists and re-evaluated until reach a consensus when the diagnoses were different. Samples were classified as NILM, atypical squamous cells of undetermined significance (ASCUS), low-grade squamous intraepithelial lesion (LSIL), high-grade squamous intraepithelial lesion (HSIL), atypical squamous cells, and it was not possible to exclude high-grade squamous intraepithelial lesions (ASC-H), squamous cervical cancer (SCC) and atypical glandular cells (AGC).

### Histology

According to the cervical cancer screening procedure, women with HR-HPV infection or abnormal cytology results may be referred for colposcopy or biopsy. When biopsy diagnosis results in ≥ HSIL, patients underwent a loop electrosurgical excision procedure cone or conization by cold knife to biopsy. Formalin (10%) was used to fix specimens, which were routinely processed for paraffin embedding. Then, 4 µm thick histological sections were cut and stained with hematoxylin and eosin using standard methods. Cervical biopsy specimens were examined and diagnosed according to the CIN system. If the review reading is inconsistent, conduct a second histological review. If two-thirds of the diagnoses are the same, the result is considered the final result.

### 16S rRNA gene-based amplicon sequencing

Genomic DNA of vaginal secretions samples was extracted by E.Z.N.A Mag-Bind Soil DNA Kit (Omega Bio-Tek, GA, USA) according to the manufacturer’s protocol. DNA samples were quantified by the Qubit 3.0 DNA Kit (Invitrogen, Waltham, MA, USA) and transferred to Sangon Biotech Testing Center (Shanghai, China) for high-throughput sequencing.

Bacterial DNA was amplified by 16S V4F primers (5′ CCTACGGGNGGCWGCAG 3′) and 16S V4R primers (5′ GACTACHVGGGTATCTAATCC 3′) complementary to the V4 region of 16S rRNA gene. This variable region has been verified can accurately amplify and resolve DNAs of vaginal microbiome. PCR reaction system consisted of 9–12 µL of nuclease-free water, 15 µL of 2 × Hieff Robust PCR Master Mix, 5 µM of each primer and 20–30 ng of genomic DNA. The cycling conditions include: initial denaturation at 95 °C for 3 min, then 94 °C for 30 s, 45 °C for 20 s, 65 °C for 30 s, then 94 °C for 20 s, 55 °C for 20 s, 72 °C for 5 min. The samples were analyzed using Roche LightCycler 480 PCR system (Roche, Switzerland).

Purified PCR products were accurately quantified by Qubit 3.0 Fluorometric High-Sensitivity dsDNA Assay (Invitrogen, Waltham, MA, USA) and then constructed library by KAPA LTP Library Kit (Kapa Biosystems, USA). High-throughput sequencing of 2 × 300 paired-end reads was performed on an Illumina MiSeq platform (Illumina, California, USA) at Sangon Biotech (Shanghai, China). Raw FASTQ files were obtained and merged.

The version of mother was v1.30.1. SILVA release 132 was used as database. R v3.6.3 was used for statistical analyses. The similarity truncation rate of the operational taxonomic units cluster was 97%. The diversity analysis of the sample (α or β diversity) could indicate the diversity or abundance of microbial communities, including the Chao and ACE (http://www.mothur.org/wiki/Chao; http://www.mothur.org/wiki/Ace) indices, for calculating the abundance of community distribution were used. Furthermore, the Shannon and Simpson (http://www.mothur.org/wiki/Shannon; http://www.mothur.org/wiki/Simpson) indices were used to calculate the diversity of community distribution. Biomarker discovery analysis was carried out by the LEfSe tool and LDA scores higher than 2.0 were considered statistically significant.

### Statistical analysis

The measurement data were counted as mean ± standard deviation in this study. The significance of BV associated with the states of HPV infection or cervical lesions was assessed by Chi-squared test or Fisher’s exact test. The data were calculated using the IBM SPSS statistical package version 22.0 (IBM, Corporation, Armonk, USA) in this study. The significance level was set at a two-tailed p-value < 0.05.

### Ethics approval and consent to participate

The study was approved approved by the Ethics Committee of Fujian Maternity and Child Health Hospital (2020KY015).

## Data Availability

All data generated or analysed during this study are included in this published article.

## Abbreviations

ASCUS: Atypical squamous cells of undetermined significance
AV: Aerobic vaginitis
BV: Bacterial vaginosis
CIN: Cervical intraepithelial neoplasia
CST: Community state type
CV: Cytolytic vaginosis
GV: *Gardnerella vaginalis*
H_2_O_2_: Hydrogen peroxide
HPV: Human papillomavirus
HR-HPV: High-risk human papillomavirus
HSIL: High-grade squamous intraepithelial lesion
LE: Leukocyte esterase
LEfSe: Linear discriminant analysis effect size
TLRs: Toll-like receptors
LSIL: Low-grade squamous intraepithelial lesion
NILM: Negative for intraepithelial lesion or malignancy
TV: Trichomonas vaginitis
TCT: Thinprep cytologic test
OTU: Operational-Taxonomy Units
RT-qPCR: Real-time quantitative PCR
SNA: Sialidase
VVC: Vulvovaginal candidiasis
